# Role of Art Therapy in the Promotion of Mental Health: A Critical Review

**DOI:** 10.7759/cureus.28026

**Published:** 2022-08-15

**Authors:** Apoorva Shukla, Sonali G Choudhari, Abhay M Gaidhane, Zahiruddin Quazi Syed

**Affiliations:** 1 Preventive Medicine, School of Epidemiology and Public Health, Datta Meghe Institute of Medical Sciences, Wardha, IND; 2 Community Medicine, Jawaharlal Nehru Medical College, Datta Meghe Institute of Medical Sciences, Wardha, IND

**Keywords:** health promotion, mood disorders, mental health, schizophrenia, art-therapy

## Abstract

Art therapy is used most commonly to treat mental illnesses and can aid in controlling manifestations correlated with psychosocially challenging behaviours, slowing cognitive decline, and enhancing the quality of life. Art therapy can help people express themselves more freely, improve their mental health, and improve interpersonal relationships. The basis of art therapy is established on the idea that people can recover and feel better via artistic expression. This review examines the current research on how active participation in the arts might improve mental health. A detailed literature search was carried out utilizing essential databases such as PubMed, the WHO's mental health database, and Google and Google Scholar. This review study looks into research done on art therapy and its potential advantages for adult mental health rehabilitation. It focuses on visual art therapy since it’s a key to reducing variation within the "creative arts" and defines the peculiar elements and effectiveness of art therapy used by mental health services. It was found that the use of art therapy as an adjunct treatment showed improved mental health in patients.

## Introduction and background

Mental health has recently been identified as a serious public health concern. Mental diseases encompass symptoms, from minor anxiety to severe forms like behavioural abnormalities [1]. Art therapy refers to various treatments, such as theatre therapy, dance movement psychotherapy, body psychotherapy, music therapy, and drawing, painting and craft therapy [2]. Art therapy uses artistic means to treat mental illnesses and improve mental health. Art has become a significant element of the therapeutic sector and is used in several recovery and treatment procedures [3]. Art therapy uses integrative techniques to captivate the soul, body and mind in ways that verbal expression alone doesn't appear to [1,4,5]. Art therapy is gaining popularity in mental health settings because it provides a recovery-oriented, person-centred approach that includes emotional, spiritual, social needs and clinical demands [6].

This narrative review is undertaken with an objective to look at the existing research on art therapies and their possible benefits in mental health rehabilitation. The review accesses the effect on the patient's creative, nonverbal, emotional and structural qualities after using art therapy. It is essential to study the contribution of art therapy to mental disorders to obtain further insights into its functioning mechanism. This helps to gain self-expression, self-awareness, learning and personal development, as well as improve contact, communication, and interaction with other people. The review focuses on visual art therapy to reduce heterogeneity and define the specific qualities of art therapy used by mental health services [7,8].

This review will be helpful to psychiatrists, psychologists, mental health nurses, peer workers, social workers, therapists, occupational therapists, mental health recovery and rehabilitation workers, and other mental health professionals. This will be of use to public health professionals and epidemiologists as they research appropriate solutions to mental health concerns that affect people's physical and social well-being, making mental health an essential part of accomplishing public health goals.

## Review

Global silhouette

Mental and behavioural problems are responsible for 12% of the global disease burden [9]. According to the World Health Organization (WHO), India's mental health burden is 2443 disability-adjusted life years (DALYs) per one lakh population, with a 21.1 age-adjusted suicide rate per one lakh population [1,9,10]. Two out of three people with psychosis worldwide do not receive specialist mental health care [1]. The WHO stated in World Health Report 2001 that "just a small proportion" of the over 45 million people worldwide who suffer from mental and behavioural problems are appropriately cared for [9].

The causes of mental illness are numerous and complicated. They differ from one situation to another [11]. The economic loss caused by mental health disorders is estimated to be USD 1.03 trillion between 2012 and 2030 [1]. Despite technological advances, the puzzle of mental disease causation remains a complex interaction of the environment, brain and mind [11].

Mechanism of art therapy

A thorough literature analysis was conducted using essential databases such as PubMed, WHO's Mental Health database and an expanded search using sources such as Google and Google Scholar to find relevant articles. Articles specifically included art therapy intervention that deals with painting, drawing and craft. Articles included for review were from 2008 to 2021. Additional filters, such as free full text, were applied to all research. Furthermore, the reference lists and publications' citations were reviewed for other relevant sources. On examining the title and abstract, final inclusion was determined. This was done to eliminate mismatches with the review topic. The keywords used for advanced search were art-therapy, art, craft, drawing, painting and mood disorders, depression, common mental disorders, anxiety disorders, dissociative disorders, depression, schizophrenia, dementia, and Alzheimer's.

Looking back into history, thousands of years ago, people were practising and dependent on the arts for self-expression, healing and communication [3,12]. However, art therapy did not become a formalized curriculum until 1940. Doctors began to notice that people with a mental illness frequently enunciate themselves through drawings and other artworks. This inspired many to consider using art as a technique to heal [3,12]. It also had a high level of patient acceptance [7]. The scope of kinaesthetic, sensory, perceptual, and symbolic communication motivates the uncommon receptive and expressive communication modes that can function beyond language's limitations [1].

Some examples of art therapy used in mental health treatment include practising art with attention to skill development and mastery, studio art making, individual art making, program supervised and structured art groups, art psychotherapy, and personal art making with a healing goal [2,7]. Community-based art-making can enhance mental well-being. Individuals can increase their sense of value and self-esteem by using visual and symbolic expressions in art therapy. This makes it possible for people with mental illnesses to interact with one another and grow their social networks [7].

Art therapists plan sessions to achieve therapeutic goals and objectives by selecting suitable materials and interventions for their clients. They engage in creative processes to support their patients' growth to increase insight, decrease stress, heal trauma, increase cognitive, memory, and neurosensory capacities, improve interpersonal relationships and achieve a sense of self-fulfillment [13].

Common mental disorders

A category of diseases known as common metal disorders (CMDs) is a functional clinical classification for "deeper psychological discomfort experiences". The International Classification of Diseases, 10th Revision (ICD-10) classification of CMDs for primary healthcare includes depression, generalized anxiety, mixed anxiety and depression, adjustment disorder, dissociative disorder, unexplained somatic symptoms, neurasthenia, sleep disorders, phobic disorder and panic disorder [14].

Fear and anxiety are natural feelings for everyone. Still, anxiety builds up over time for those with anxiety disorders. Eventually, it does not commensurate with the actual threat or risk and becomes permanent [15]. Anxiety disorders are linked to problems with self-control [16].

Art therapy and mood disorders

Manic episodes, depressive episodes, bipolar disorder, recurrent depressive disorder and persistent mood disorder are all classified as mood disorders in the ICD-10 [14]. About 5% of adults worldwide suffer from depression. As stated by the WHO, women are more affected by depression than men. Depression significantly contributes to the global disease burden and can lead to suicide [17]. Major depressive disorder can have physical and mental side effects that affect one's overall physical well-being and quality of life. Only about half of individuals with depressive illnesses get their needed help [18]. The elderly, low-income and minority populations are more likely to be denied care. In the treatment of depression, pharmacotherapy is widely used, especially in moderate to severe instances [18,19].

Anxiety and mood disorder management has greatly succeeded in terms of understanding and treatment in the last two decades. Despite identifying unique processes, many believe creative expression can considerably improve mental health in clinical and community populations [7,16].

The evidence that shows how art therapy worked on people with mood disorders depicts different results. In a randomized control trial (RCT) by Ciasca et al., art therapists provided verbal advice as an introduction to the artistic procedure to help participants connect with the feelings and images associated with the theme being discussed. Patients utilized the resources available to deal with their problems. Techniques included weaving, collage, clay modelling, drawing and painting [18]. This randomized, single-blind trial found that introducing art-therapy intervention in patients with stable and pharmacologically treated major depressive disorder improved depression and anxiety symptoms. In another study, 118 participants 18-65 years of age were randomly assigned to intervention and standard therapy groups. No significant differences existed at baseline, and both groups demonstrated gains during the follow-up [20].

The effectiveness of art therapy on anxiety-related symptoms, anxiety severity, quality of life, and emotion regulation were examined. Studies have investigated the aspects that influence a treatment. Participants who had been diagnosed with generalized anxiety disorder, social anxiety disorder or panic disorder and had mild to moderate anxiety symptoms were introduced to art-therapy intervention. The study comprised a variety of creative exercises selected from a list of art-therapy activities. Because art therapy is a highly customized treatment, no set treatment procedure was used. Three types of exercises were offered: clay work, painting and drawing. The therapists documented the intricacies of the therapeutic approaches. Researchers then verified whether the deployed activities helped achieve the treatment objectives. According to the RCT, emotion regulation is a factor that causes anxiety reduction via art therapy [16]. The most significant gains in emotion regulation were better emotion acceptance and goal-oriented actions [8].

Art therapy and schizophrenia

Schizophrenia is a serious mental illness that has an adverse effect on 1 in 300 persons worldwide or about 24 million people. At least one in three people with schizophrenia will be able to recover, thanks to a variety of efficient treatment options [21]. Positive formal thought disorder, delusions, hallucinations and consistently unusual behaviour are characteristics of positive schizophrenia. Avolition, anhedonia, alogia, avolition, and affective flattening are the hallmarks of negative schizophrenia. In mixed schizophrenia, either both negative and positive symptoms are noticeable, or neither is noticeable [22].

Along with "positive" symptoms like delusions and hallucinations, many people have also encountered motivation loss, diversified energy levels and diminished concentration [22]. Some investigators have also attempted to investigate the productivity of schizophrenic patients using group art therapy as a complementary treatment. Patients were provided with various creative tools and encouraged to convey themselves freely. Art therapists often took a positive attitude toward empathizing with and encouraging their patients [23].

Various trials on the contribution of art therapy in schizophrenia have showed effective outcomes. The preliminary outcome showed identical results in the trial done by Crawford et al. to see if there were any benefits of group art therapy for schizophrenic patients [23]. Referring persons with schizophrenia to group art therapy, as done in this study, did not affect overall functioning, mental health or other health-associated outcomes [23]. However, in another pilot study, the art-therapy interventions aimed to assist the artist's process and understanding of the image. The patients who got art therapy showed a significant average decline in positive and negative manifestations of schizophrenia than patients who received standard care [24]. It implies that when art therapy was recommended to people with schizophrenia, they had greater emotional awareness scores at the end of the intervention period than patients treated with standard treatment. At post-treatment and follow-up, the art-therapy group had scarcely any positive manifestations of schizophrenia than the control group [24]. On the other hand, the most extensive, published three-arm RCT on art therapy (Multicenter Study of Art Therapy in Schizophrenia: Systematic Evaluation, or MATISSE trial) found no additional benefit in the global assessment of functioning in positive and negative manifestation scores. In this study, 417 schizophrenic patients were randomly allocated to art therapy and it did not enhance patient outcomes [2,23].

Art therapy and dementia

Dementia is described by a decline in the ability to remember, think or do other cognitive operations. Dementia comes in various forms, and multiple conditions can cause it [25]. More than 55 million people worldwide suffer from dementia, with around 10 million new cases diagnosed every year [26]. Mixed dementia is a condition in which different forms of dementia emerge in the brain. Alzheimer's disease is the leading cause of dementia and accounts for 60%-80% of cases. Dementia does not occur naturally as part of the ageing process. Damage to brain cells leads to a loss in the capacity to communicate, impacting thought, behaviour and feelings [25-27].

Art therapy, dance or movement therapy, music therapy and reminiscence therapy have all been studied as non-pharmacological treatments for Alzheimer's [27]. Studies have shown multidisciplinary cognitive rehabilitation in patients with mild Alzheimer's [28]. Art therapy was used as one of the activities for cognitive rehabilitation. Memory training, computer-assisted mental stimulation, expressive activities (drawing, verbal expression, writing), physiotherapy and physical training were all employed. As a consequence, patients' quality of life improved [29]. Participation in art interventions involving dance, expressive writing, music, theatre and visual arts was studied for their therapeutic and psychological impacts [30].

Richards et al. introduced art-therapy exercises to the intervention group for one and a half hours in their study. The activities included hat decoration, collage, embossing, painting, pottery, photography and printmaking. Each week, the participants developed an art product based on the instructions. Art therapy has been shown to boost self-esteem by reinforcing emotions of self-worth or competence. Finally, following the two-month course, participants continued to create artwork independently, resulting in a stronger sense of accomplishment and enhanced self-esteem [31,32].

Art-based practices and their benefits

According to a literature search, art therapy treats various mental problems. Research is currently looking into the contribution of art therapy in multiple areas, including depression, dementia, schizophrenia and psychosis. More extensive studies have examined topics like overall wellness or everyday anxiety [24,33]. It has been proposed that art can help people increase their self-esteem by providing abilities that can be acquired and mastered. Creating art, whether one becomes an artist or not, is inspiring. It develops inventive talents and an awareness of work planning and execution. It fosters the sense of self-sufficiency and the deep satisfaction of using one's artistic ability and mental capacity to generate outcomes one values and desires to share further.

Participating in creative activities can help people cope with stress and despair and alleviate the burden of chronic mental illnesses [7]. Many cultures have accepted the idea that artistic expression may considerably aid in the healing process. Throughout history, people have used paintings, storytelling, dances, yoga, and chants as healing rituals [7,34,35]. Over the last decade, health psychologists have carefully examined how art therapy help heal emotional traumas, enhance awareness of oneself and others, establish self-reflection capacity, reduce mental manifestations and transform behaviours and way of thinking.

Eisner best expresses this view of art education: "There are few things more problematic than a white sheet of paper or a lump of clay". Artists must create an art form out of these components. They must battle to understand how to feel about their efforts as they go and how to use their intelligence and experience to alter and improve them so that the ultimate obstacle is appropriately managed [36]. To put it another way, the process of creating an artwork is a continuous challenge to solve, and it involves numerous cognitive, emotional and physical factors.

Art therapy is also employed as a treatment modality in cancer patients, autism, HIV patients, Alzheimer's disease, COVID-19, dementia and Parkinson’s disease [34,37,38]. Art activities other than art and craft are music therapy, cognitive behavioural therapy, behavioural therapy, occupational therapy, clay modelling, and psychodynamic psychotherapy. These therapies also provide evidence about their effect on mental health. Unexplored art activities might provide additional proof of the impact on mental health, which includes fiction writing, sketching, interpretive dancing and photography [30,39].

## Conclusions

The basic components of art-therapy interventions in the real world are hard to determine because there are currently no effective strategies for therapeutic, engaging, sensory art-therapy interventions. There is inadequate data to support the effectiveness of art therapy, and hence, more well-powered, high-quality trials with relevant outcome measures are required and more research is needed on the subject. Also, results of an intervention are not solely dependent on art therapy, as it is used along with pharmacotherapy. This made comparing all of the results difficult. The research comprising a small number of people makes it impossible to know how precise the results are, making it complicated to predict if the results will be the same in larger groups of people.

It is vital to raise awareness and mobilize support for mental health. Mental health concerns must be treated as soon as possible. Comprehensive measures for promotion, prevention, treatment and rehabilitation can be implemented through government approaches. Policymakers should be urged to enhance access to cost-effective treatment for prevailing mental illnesses in primary healthcare settings.
